# The top 100 most‐cited publications on osteochondral lesions of the ankle: A bibliometric analysis

**DOI:** 10.1002/jeo2.70850

**Published:** 2026-07-20

**Authors:** Jari Dahmen, Juliëtte H.M. Pijnacker, Youichi Yasui, Faridi S. Jamaludin, Masato Takao, John G. Kennedy, Gino M. M. J. Kerkhoffs, International Society for Cartilage Repair of the Ankle (ISCRA) Study Group, Nasef M. N. Abdelatif, Chayanin Angthong, James D. F. Calder, Kaj. S. Emanuel, Erik Ferkel, Richard Ferkel, Elze Geurts, Arianna L. Gianakos, MaCalus V. Hogan, Julian J. Hollander, Pieter d'Hooghe, Stephen Kearns, Christopher D. Murawski, Tomoyuki Nakasa, Helder Pereira, Quinten G. H. Rikken, Jason A. H. Steman, James W. Stone, Sjoerd Stufkens, Jozef J. M. Suskens, Federico G. Usuelli, Markus Walther, Alastair Younger

**Affiliations:** ^1^ Department of Orthopedic Surgery and Sports Medicine Amsterdam Movement Sciences, Amsterdam UMC, Location AMC University of Amsterdam Amsterdam the Netherlands; ^2^ Academic Center for Evidence based Sports medicine (ACES), Amsterdam UMC Amsterdam the Netherlands; ^3^ Amsterdam Collaboration for Health and Safety in Sports (ACHSS), International Olympic Committee (IOC) Research Center, Amsterdam UMC Amsterdam the Netherlands; ^4^ Department of Orthopaedic Surgery Teikyo University School of Medicine Tokyo Japan; ^5^ Amsterdam UMC Location University of Amsterdam, Medical Library AMC Amsterdam the Netherlands; ^6^ Clinical and Research Institute for Foot and Ankle Surgery, Jujo Hospital Kisarazu Chiba Japan; ^7^ Department of Orthopedic Surgery NYU Langone Health New York New York USA

**Keywords:** analysis, ankle cartilage, bibliometric, citations, osteochondral

## Abstract

**Purpose:**

The purpose was to identify the 100 most‐cited scientific publications in ankle cartilage and provide an analysis of their main characteristics.

**Methods:**

The Web of Science (Clarivate) database was searched in April 2025 to obtain data and metrics on ankle cartilage research. The search list was sorted by the number of citations, and articles were included or excluded based on their relevance to ankle cartilage research. The information extracted for each article included the publication year, citation count, author's name, country of origin, journal name, article type, citation density and the level of evidence.

**Results:**

The 100 studies generated a total of 17,208 citations, with an average of 172 citations per article. The most‐cited article was cited 489 times. The 100 studies included in this analysis were published between 1979 and 2018. In total, these articles represented 13 different countries of origin. The United States represented 38 of the 100 articles. The Netherlands, Italy and Germany followed with nine articles, and South Korea with seven articles. The most prevalent study designs were case series, and the most prevalent level of evidence was Level IV (69 studies).

**Conclusions:**

The present research concludes that the 100 most‐cited publications in cartilage lesions of the ankle research were cited a total of 17,208 times. Case series and cohort studies were among the most frequently used study designs with an overall relatively low‐level of evidence. The present work may function as a reference standard to help direct orthopaedic providers to the 100 most‐cited studies in cartilage lesions of the ankle.

**Level of Evidence:**

N/A.

**Abbreviations**
N/A: not applicable
OCL: osteochondral lesion
OLT: osteochondral lesion of the talus
RCT: randomized controlled trial

## INTRODUCTION

Osteochondral lesions (OCLs) of the ankle are often a result of a preceding trauma to the ankle. In this process, an ankle fracture or sprain may initially result in invisible cartilage damage to the ankle [18, 22, 71]. Subsequently, the lesion can progress into an OCL of the talus or distal tibia [20] and pose a clinical challenge [109]. Historically, treatment options have included non‐operative treatment, minimally invasive surgical techniques with bone marrow stimulation, to more advanced and invasive procedures, such as autologous chondrocyte implantation and osteo(chondral) autografting [19, 60]. As our understanding of cartilage repair has evolved over the past few decades, there has been an increasing interest in biological and regenerative approaches aimed at improving longer‐term outcomes [9]. Recent advancements are emphasizing personalized approaches based on lesion size, location, morphology and patient‐specific factors [17, 85].

A growing body of evidence on ankle cartilage lesions has resulted in a continuously expanding volume of scientific literature, often making it challenging for clinicians and researchers to identify the most impactful studies over time [110, 111]. Bibliometric studies have been conducted in other orthopaedic subspecialties, including in knee cartilage repair and osteotomy procedures around the knee [77]. However, a comprehensive citation analysis specific to ankle cartilage lesions has not previously been performed. Such tools may be helpful in highlighting influential publications that have shaped the current knowledge and guided daily clinical practice. Moreover, such an analysis can assist in outlining future research directions.

Consequently, the primary purpose of the present study was to identify the one hundred most‐cited publications on cartilage lesions of the ankle and analyze their key characteristics. We hypothesized that a majority of the most‐cited studies were published in the last 20 years, reflecting the increasing focus on cartilage repair and regeneration in ankle surgery.

## METHODS

A methodology similar to the work by Ollivier et al. [77] was used as the basis for the present manuscript. An institutional review board was deemed unnecessary for the present study, given that the publication data were readily available in the public domain. Data was retrieved from the Web of Science (Clarivate) database using Boolean queries [49, 101].

### Search

The initial database search took place on 2 April 2025, incorporating various Boolean search terms to capture all possible iterations of cartilage and osteochondral lesions of the ankle. The search strategy that was used was the following: ALL = (‘osteochondral lesion*’ OR ‘osteochondral defect*’ OR ‘cartilage lesion*’)) AND (ALL = (ankle* OR talus)). The search was carried out by a certified medical librarian (FJ) without any limitations on date of publication, journal, or country of origin. Solely publications in English were included. The literature was searched from inception to 2 April 2025. The search strategy was pilot‐tested to ensure adequate sensitivity with the help of a librarian.

Subsequently, the list of publications was organized by the total number of citations in descending order. The title and abstract of each publication were reviewed by one author (J.D.) to determine relevance to cartilage lesions of the ankle. To qualify for selection, the publication had to present information on cartilage lesions of the ankle, including chondral and osteochondral lesions of the talus and distal tibial plafond. Publications that did not meet these criteria were not included. If the publication were mentioned only briefly without a primary focus on chondral or osteochondral lesions of the ankle, it was excluded. Articles were excluded if a mixture of the ankle and other joints was researched. Studies on cartilage lesions of the ankle, primarily focusing on haemophilia, were excluded. If, after full‐text review, uncertainty remained regarding eligibility, two authors (J.D. and J.H.M.P.) jointly evaluated the study to reach a consensus on inclusion or exclusion. If consensus could not be achieved, a third author (G.M.M.J.K.) was consulted to make the final decision.

### Data extraction

The author's country of origin, number of citations, journal title, year of publication and study design (laboratory study, animal study, review article, descriptive study, case report, case series, cohort study, case–control and randomized controlled trial [RCT]) were extracted. In addition, the primary thematic focus of each publication was recorded and categorized into one of five predefined groups: (1) treatment, (2) reviews/overviews, (3) paediatric or juvenile osteochondral lesions of the talus (OLT), (4) radiology/imaging and (5) aetiology/pathophysiology. The level of evidence with the article's relative risk of bias was determined based on guidelines published in The Journal of Arthroscopy and Related Surgery [51]. The study design and level of evidence were classified by the consensus opinion between two authors (J.D. and J.H.M.P.). If a consensus could not be obtained, the senior author (G.M.M.J.K.) was consulted for the final decision. The final list of the 100 most‐cited articles was then organized based on total citations and presented in descending order. Similar to Ollivier et al. [77], citation density was calculated by dividing the total number of citations by the years since the paper was published. If two articles had the same position based on total citations, the higher citation density was placed first.

## RESULTS

**Figure 1:**
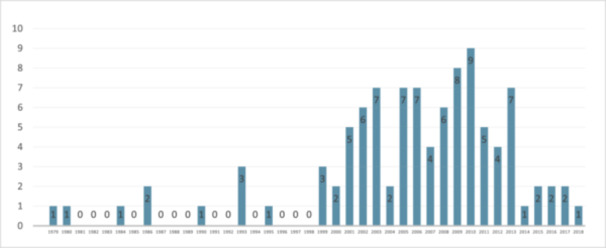
The number of most‐cited 100 cartilage lesions of the ankle publications per year.

A total of 194 of the most‐cited articles were reviewed to reach the top 100 most‐cited studies that met the designed inclusion criteria. The 100 most‐cited articles on cartilage lesions of the ankle were published between 1979 and 2018 (Figure 1). The cumulative number of citations for these 100 publications totalled 17,208, which resulted in an average of 172 citations per paper and ranged from 88 to 489 (Table 1). The most‐cited publication had 489 citations. It was also the most citation‐dense, averaging 33 citations per year. Table 1 provides an overview of the top 100 most‐cited articles.

**Table 1:** The top 100 cited cartilage lesions of the ankle publications.

Rank	Article	No. of citations	Publication year	Study design	Citation density	Level of evidence	Country
1	Zengerink M, Struijs PA, Tol JL, van Dijk CN. Treatment of osteochondral lesions of the talus: a systematic review. Knee Surg Sports Traumatol Arthrosc. 2010 [116]	489	2010	Review article	33	IV	Netherlands
2	Canale ST, Belding RH. Osteochondral lesions of the talus. J Bone Joint Surg Am. 1980 [10]	415	1980	Case series	9	IV	United States
3	Chuckpaiwong B, Berkson EM, Theodore GH. Microfracture for osteochondral lesions of the ankle: outcome analysis and outcome predictors of 105 cases. Arthroscopy. 2008 [14]	389	2008	Cohort study	23	II	United States
4	Hangody L, Vásárhelyi G, Hangody LR, Sükösd Z, Tibay G, Bartha L, Bodó G. Autologous osteochondral grafting‐‐technique and long‐term results. Injury. 2008 [46]	379	2008	Cohort study	22	II	Hungary
5	Raikin SM, Elias I, Zoga AC, Morrison WB, Besser MP, Schweitzer ME. Osteochondral lesions of the talus: localization and morphologic data from 424 patients using a novel anatomical grid scheme. Foot Ankle Int. 2007 [25]	374	2007	Case series	21	IV	United States
6	Choi WJ, Park KK, Kim BS, Lee JW. Osteochondral lesion of the talus: is there a critical defect size for poor outcome? Am J Sports Med. 2009 [12]	364	2009	Case series	23	IV	South Korea
7	Ferkel RD, Zanotti RM, Komenda GA, Sgaglione NA, Cheng MS, Applegate GR, Dopirak RM. Arthroscopic treatment of chronic osteochondral lesions of the talus: long‐term results. Am J Sports Med. 2008 [26]	359	2008	Case series	21	IV	United States
8	Tol JL, Struijs PA, Bossuyt PM, Verhagen RA, van Dijk CN. Treatment strategies in osteochondral defects of the talar dome: a systematic review. Foot Ankle Int. 2000 [104]	322	2000	Review article	13	IV	Netherlands
9	Hepple S, Winson IG, Glew D. Osteochondral lesions of the talus: a revised classification. Foot Ankle Int. 1999 [50]	303	1999	Cohort study	12	III	United kingdom
10	Hangody L, Kish G, Módis L, Szerb I, Gáspár L, Diószegi Z, Kendik Z. Mosaicplasty for the treatment of osteochondritis dissecans of the talus: two to seven year results in 36 patients. Foot Ankle Int. 2001 [45]	297	2001	Case series	12	IV	Hungary
11	Loomer R, Fisher C, Lloyd‐Smith R, Sisler J, Cooney T. Osteochondral lesions of the talus. Am J Sports Med. 1993 [63]	294	1993	Case series	9	IV	Canada
12	van Dijk CN, Reilingh ML, Zengerink M, van Bergen CJ. Osteochondral defects in the ankle: why painful? Knee Surg Sports Traumatol Arthrosc. 2010 [112]	286	2010	Review article	19	V	Netherlands
13	Gobbi A, Francisco RA, Lubowitz JH, Allegra F, Canata G. Osteochondral lesions of the talus: randomized controlled trial comparing chondroplasty, microfracture, and osteochondral autograft transplantation. Arthroscopy. 2006 [37]	283	2006	RCT	15	II	Italy
14	O'Loughlin PF, Heyworth BE, Kennedy JG. Current concepts in the diagnosis and treatment of osteochondral lesions of the ankle. Am J Sports Med. 2010 [76]	276	2010	Review article	18	V	United States
15	Saxena A, Eakin C. Articular talar injuries in athletes: results of microfracture and autogenous bone graft. Am J Sports Med. 2007 [89]	265	2007	Case series	15	IV	United States
16	Verhagen RA, Struijs PA, Bossuyt PM, van Dijk CN. Systematic review of treatment strategies for osteochondral defects of the talar dome. Foot Ankle Clin. 2003 [114]	262	2003	Review article	12	IV	Netherlands
17	Giannini S, Buda R, Vannini F, Cavallo M, Grigolo B. One‐step bone marrow‐derived cell transplantation in talar osteochondral lesions. Clin Orthop Relat Res. 2009 [33]	245	2009	Case series	15	IV	Italy
18	Reddy S, Pedowitz DI, Parekh SG, Sennett BJ, Okereke E. The morbidity associated with osteochondral harvest from asymptomatic knees for the treatment of osteochondral lesions of the talus. Am J Sports Med. 2007 [84]	237	2007	Case series	13	IV	United States
19	Pritsch M, Horoshovski H, Farine I. Arthroscopic treatment of osteochondral lesions of the talus. J Bone Joint Surg Am. 1986 [81]	236	1986	Case series	6	IV	Israel
20	Kumai T, Takakura Y, Higashiyama I, Tamai S. Arthroscopic drilling for the treatment of osteochondral lesions of the talus. J Bone Joint Surg Am. 1999 [58]	235	1999	Case series	9	IV	Japan
21	Taga I, Shino K, Inoue M, Nakata K, Maeda A. Articular cartilage lesions in ankles with lateral ligament injury. An arthroscopic study. Am J Sports Med. 1993 [98]	233	1993	Case series	7	IV	Japan
22	Mei‐Dan O, Carmont MR, Laver L, Mann G, Maffulli N, Nyska M. Platelet‐rich plasma or hyaluronate in the management of osteochondral lesions of the talus. Am J Sports Med. 2012 [68]	228	2012	RCT	18	II	Israel
23	Murawski CD, Kennedy JG. Operative treatment of osteochondral lesions of the talus. J Bone Joint Surg Am. 2013 [72]	221	2013	Review article	18	V	United States
24	Verhagen RA, Maas M, Dijkgraaf MG, Tol JL, Krips R, van Dijk CN. Prospective study on diagnostic strategies in osteochondral lesions of the talus. Is MRI superior to helical CT? J Bone Joint Surg Br. 2005 [113]	220	2005	Cohort study	11	II	Netherlands
25	Valderrabano V, Leumann A, Rasch H, Egelhof T, Hintermann B, Pagenstert G. Knee‐to‐ankle mosaicplasty for the treatment of osteochondral lesions of the ankle joint. Am J Sports Med. 2009 [105]	215	2009	Case series	13	IV	Switzerland
26	Ramponi L, Yasui Y, Murawski CD, Ferkel RD, DiGiovanni CW, Kerkhoffs GMMJ, Calder JDF, Takao M, Vannini F, Choi WJ, Lee JW, Stone J, Kennedy JG. Lesion Size Is a Predictor of Clinical Outcomes After Bone Marrow Stimulation for Osteochondral Lesions of the Talus: A Systematic Review. Am J Sports Med. 2017 [83]	215	2017	Review article	27	IV	United States
27	Gross AE, Agnidis Z, Hutchison CR. Osteochondral defects of the talus treated with fresh osteochondral allograft transplantation. Foot Ankle Int. 2001 [39]	207	2001	Case series	9	IV	Canada
28	Taranow WS, Bisignani GA, Towers JD, Conti SF. Retrograde drilling of osteochondral lesions of the medial talar dome. Foot Ankle Int. 1999 [102]	203	1999	Case series	8	IV	United States
29	Giannini S, Vannini F. Operative treatment of osteochondral lesions of the talar dome: current concepts review. Foot Ankle Int. 2004 [35]	201	2004	Review article	10	V	Italy
30	De Smet AA, Fisher DR, Burnstein MI, Graf BK, Lange RH. Value of MR imaging in staging osteochondral lesions of the talus (osteochondritis dissecans): results in 14 patients. AJR Am J Roentgenol. 1990 [21]	199	1990	Case series	6	IV	United States
31	Mintz DN, Tashjian GS, Connell DA, Deland JT, O'Malley M, Potter HG. Osteochondral lesions of the talus: a new magnetic resonance grading system with arthroscopic correlation. Arthroscopy. 2003 [70]	196	2003	Case series	9	IV	United States
32	Giannini S, Buda R, Vannini F, Di Caprio F, Grigolo B. Arthroscopic autologous chondrocyte implantation in osteochondral lesions of the talus: surgical technique and results. Am J Sports Med. 2008 [34]	196	2008	Case series	12	IV	Italy
33	Robinson DE, Winson IG, Harries WJ, Kelly AJ. Arthroscopic treatment of osteochondral lesions of the talus. J Bone Joint Surg Br. 2003 [86]	195	2003	Case series	9	IV	United Kingdom
34	Schuman L, Struijs PA, van Dijk CN. Arthroscopic treatment for osteochondral defects of the talus. Results at follow‐up at 2 to 11 years. J Bone Joint Surg Br. 2002 [92]	188	2002	Case series	8	IV	Netherlands
35	Scranton PE Jr, Frey CC, Feder KS. Outcome of osteochondral autograft transplantation for type‐V cystic osteochondral lesions of the talus. J Bone Joint Surg Br. 2006 [93]	187	2006	Case series	10	IV	United States
36	Gautier E, Kolker D, Jakob RP. Treatment of cartilage defects of the talus by autologous osteochondral grafts. J Bone Joint Surg Br. 2002 [27]	174	2002	Case series	8	IV	Switzerland
37	Athanasiou KA, Niederauer GG, Schenck RC Jr. Biomechanical topography of human ankle cartilage. Ann Biomed Eng. 1995 [3]	172	1995	Laboratory study	6	IV	United States
38	Al‐Shaikh RA, Chou LB, Mann JA, Dreeben SM, Prieskorn D. Autologous osteochondral grafting for talar cartilage defects. Foot Ankle Int. 2002 [1]	172	2002	Case series	7	IV	United States
39	Giannini S, Buda R, Grigolo B, Vannini F. Autologous chondrocyte transplantation in osteochondral lesions of the ankle joint. Foot Ankle Int. 2001 [31]	171	2001	Case series	7	IV	Italy
40	Becher C, Thermann H. Results of microfracture in the treatment of articular cartilage defects of the talus. Foot Ankle Int. 2005 [8]	170	2005	Case series	9	IV	Germany
41	Whittaker JP, Smith G, Makwana N, Roberts S, Harrison PE, Laing P, Richardson JB. Early results of autologous chondrocyte implantation in the talus. J Bone Joint Surg Br. 2005 [115]	162	2005	Case series	8	IV	United Kingdom
42	Raikin SM. Fresh osteochondral allografts for large‐volume cystic osteochondral defects of the talus. J Bone Joint Surg Am. 2009 [82]	159	2009	Case series	10	IV	United States
43	Valderrabano V, Miska M, Leumann A, Wiewiorski M. Reconstruction of osteochondral lesions of the talus with autologous spongiosa grafts and autologous matrix‐induced chondrogenesis. Am J Sports Med. 2013 [106]	156	2013	Case series	13	IV	Switzerland
44	Giannini S, Buda R, Battaglia M, Cavallo M, Ruffilli A, Ramponi L, Pagliazzi G, Vannini F. One‐step repair in talar osteochondral lesions: 4‐year clinical results and t2‐mapping capability in outcome prediction. Am J Sports Med. 2013 [29]	155	2013	Case series	13	IV	Italy
45	Giannini S, Buda R, Cavallo M, Ruffilli A, Cenacchi A, Cavallo C, Vannini F. Cartilage repair evolution in post‐traumatic osteochondral lesions of the talus: from open field autologous chondrocyte to bone‐marrow‐derived cells transplantation. Injury. 2010 [30]	154	2010	Case series	10	IV	Italy
46	Paul J, Sagstetter A, Kriner M, Imhoff AB, Spang J, Hinterwimmer S. Donor‐site morbidity after osteochondral autologous transplantation for lesions of the talus. J Bone Joint Surg Am. 2009 [79]	153	2009	Case series	10	IV	Germany
47	van Bergen CJ, Kox LS, Maas M, Sierevelt IN, Kerkhoffs GM, van Dijk CN. Arthroscopic treatment of osteochondral defects of the talus: outcomes at eight to twenty years of follow‐up. J Bone Joint Surg Am. 2013 [108]	153	2013	Case series	13	IV	Netherlands
48	Stufkens SA, Knupp M, Horisberger M, Lampert C, Hintermann B. Cartilage lesions and the development of osteoarthritis after internal fixation of ankle fractures: a prospective study. J Bone Joint Surg Am. 2010 [97]	149	2010	Cohort study	10	II	Switzerland
49	Baums MH, Heidrich G, Schultz W, Steckel H, Kahl E, Klinger HM. Autologous chondrocyte transplantation for treating cartilage defects of the talus. J Bone Joint Surg Am. 2006 [7]	147	2006	Case series	8	IV	Germany
50	Kennedy JG, Murawski CD. The Treatment of Osteochondral Lesions of the Talus with Autologous Osteochondral Transplantation and Bone Marrow Aspirate Concentrate: Surgical Technique. Cartilage. 2011 [54]	143	2011	Case series	10	IV	United States
51	El‐Rashidy H, Villacis D, Omar I, Kelikian AS. Fresh osteochondral allograft for the treatment of cartilage defects of the talus: a retrospective review. J Bone Joint Surg Am. 2011 [23]	138	2011	Case series	10	IV	United States
52	Dahmen J, Lambers KTA, Reilingh ML, van Bergen CJA, Stufkens SAS, Kerkhoffs GMMJ. No superior treatment for primary osteochondral defects of the talus. Knee Surg Sports Traumatol Arthrosc. 2018 [19]	138	2018	Review article	20	IV	Netherlands
53	Baltzer AW, Arnold JP. Bone‐cartilage transplantation from the ipsilateral knee for chondral lesions of the talus. Arthroscopy. 2005 [6]	137	2005	Case series	7	IV	Germany
54	Schmid MR, Pfirrmann CW, Hodler J, Vienne P, Zanetti M. Cartilage lesions in the ankle joint: comparison of MR arthrography and CT arthrography. Skeletal Radiol. 2003 [91]	130	2003	Cohort study	6	II	Switzerland
55	Looze CA, Capo J, Ryan MK, Begly JP, Chapman C, Swanson D, Singh BC, Strauss EJ. Evaluation and Management of Osteochondral Lesions of the Talus. Cartilage. 2017 [64]	130	2017	Review article	16	V	United States
56	McCullough CJ, Venugopal V. Osteochondritis dissecans of the talus: the natural history. Clin Orthop Relat Res. 1979 [65]	126	1979	Case series	3	IV	United Kingdom
57	Kono M, Takao M, Naito K, Uchio Y, Ochi M. Retrograde drilling for osteochondral lesions of the talar dome. Am J Sports Med. 2006 [56]	126	2006	Case control	7	III	Japan
58	Lee KB, Bai LB, Yoon TR, Jung ST, Seon JK. Second‐look arthroscopic findings and clinical outcomes after microfracture for osteochondral lesions of the talus. Am J Sports Med. 2009 [62]	126	2009	Case series	8	IV	South Korea
59	Sammarco GJ, Makwana NK. Treatment of talar osteochondral lesions using local osteochondral graft. Foot Ankle Int. 2002 [88]	125	2002	Case series	5	IV	United States
60	Kreuz PC, Steinwachs M, Erggelet C, Lahm A, Henle P, Niemeyer P. Mosaicplasty with autogenous talar autograft for osteochondral lesions of the talus after failed primary arthroscopic management: a prospective study with a 4‐year follow‐up. Am J Sports Med. 2006 [57]	125	2006	Case series	7	IV	Germany
61	Giannini S, Battaglia M, Buda R, Cavallo M, Ruffilli A, Vannini F. Surgical treatment of osteochondral lesions of the talus by open‐field autologous chondrocyte implantation: a 10‐year follow‐up clinical and magnetic resonance imaging T2‐mapping evaluation. Am J Sports Med. 2009 [28]	124	2009	Case series	8	IV	Italy
62	Hannon CP, Smyth NA, Murawski CD, Savage‐Elliott I, Deyer TW, Calder JD, Kennedy JG. Osteochondral lesions of the talus: aspects of current management. Bone Joint J. 2014 [48]	124	2014	Review article	11	V	United States
63	Badekas T, Takvorian M, Souras N. Treatment principles for osteochondral lesions in foot and ankle. Int Orthop. 2013 [5]	123	2013	Review article	10	V	Greece
64	Parisien JS. Arthroscopic treatment of osteochondral lesions of the talus. Am J Sports Med. 1986 [78]	120	1986	Case series	3	IV	United States
65	Choi YS, Potter HG, Chun TJ. MR imaging of cartilage repair in the knee and ankle. Radiographics. 2008 [13]	119	2008	Review article	7	V	South Korea
66	Lahm A, Erggelet C, Steinwachs M, Reichelt A. Arthroscopic management of osteochondral lesions of the talus: results of drilling and usefulness of magnetic resonance imaging before and after treatment. Arthroscopy. 2000 [59]	118	2000	Case series	5	IV	Germany
67	Haene R, Qamirani E, Story RA, Pinsker E, Daniels TR. Intermediate outcomes of fresh talar osteochondral allografts for treatment of large osteochondral lesions of the talus. J Bone Joint Surg Am. 2012 [41]	117	2012	Case series	9	IV	Canada
68	Takao M, Ochi M, Uchio Y, Naito K, Kono T, Oae K. Osteochondral lesions of the talar dome associated with trauma. Arthroscopy. 2003 [99]	116	2003	Case series	5	IV	Japan
69	Schachter AK, Chen AL, Reddy PD, Tejwani NC. Osteochondral lesions of the talus. J Am Acad Orthop Surg. 2005 [90]	115	2005	Review article	6	V	United States
70	Scranton PE Jr, McDermott JE. Treatment of type V osteochondral lesions of the talus with ipsilateral knee osteochondral autografts. Foot Ankle Int. 2001[94]	113	2001	Case series	5	IV	United States
71	Assenmacher JA, Kelikian AS, Gottlob C, Kodros S. Arthroscopically assisted autologous osteochondral transplantation for osteochondral lesions of the talar dome: an MRI and clinical follow‐up study. Foot Ankle Int. 2001 [2]	111	2001	Case series	5	IV	United States
72	Imhoff AB, Paul J, Ottinger B, Wörtler K, Lämmle L, Spang J, Hinterwimmer S. Osteochondral transplantation of the talus: long‐term clinical and magnetic resonance imaging evaluation. Am J Sports Med. 2011 [53]	111	2011	Case series	8	IV	Germany
73	McGahan PJ, Pinney SJ. Current concept review: osteochondral lesions of the talus. Foot Ankle Int. 2010 [66]	109	2010	Review article	7	V	United States
74	Giza E, Sullivan M, Ocel D, Lundeen G, Mitchell ME, Veris L, Walton J. Matrix‐induced autologous chondrocyte implantation of talus articular defects. Foot Ankle Int. 2010 [36]	109	2010	Case series	7	IV	United States
75	Aurich M, Bedi HS, Smith PJ, Rolauffs B, Mückley T, Clayton J, Blackney M. Arthroscopic treatment of osteochondral lesions of the ankle with matrix‐associated chondrocyte implantation: early clinical and magnetic resonance imaging results. Am J Sports Med. 2011 [4]	105	2011	Case series	8	IV	Germany
76	Guney A, Akar M, Karaman I, Oner M, Guney B. Clinical outcomes of platelet rich plasma (PRP) as an adjunct to microfracture surgery in osteochondral lesions of the talus. Knee Surg Sports Traumatol Arthrosc. 2015 [40]	105	2015	RCT	11	II	Türkiye
77	Navid DO, Myerson MS. Approach alternatives for treatment of osteochondral lesions of the talus. Foot Ankle Clin. 2002 [74]	102	2002	Review article	4	V	United States
78	Polat G, Erşen A, Erdil ME, Kızılkurt T, Kılıçoğlu Ö, Aşık M. Long‐term results of microfracture in the treatment of talus osteochondral lesions. Knee Surg Sports Traumatol Arthrosc. 2016 [80]	101	2016	Case series	11	IV	Türkiye
79	Lee KB, Bai LB, Chung JY, Seon JK. Arthroscopic microfracture for osteochondral lesions of the talus. Knee Surg Sports Traumatol Arthrosc. 2010 [61]	100	2010	Case series	7	IV	South Korea
80	Niemeyer P, Salzmann G, Schmal H, Mayr H, Südkamp NP. Autologous chondrocyte implantation for the treatment of chondral and osteochondral defects of the talus: a meta‐analysis of available evidence. Knee Surg Sports Traumatol Arthrosc. 2012 [75]	100	2012	Review article	8	IV	Germany
81	Görmeli G, Karakaplan M, Görmeli CA, Sarıkaya B, Elmalı N, Ersoy Y. Clinical Effects of Platelet‐Rich Plasma and Hyaluronic Acid as an Additional Therapy for Talar Osteochondral Lesions Treated with Microfracture Surgery: A Prospective Randomized Clinical Trial. Foot Ankle Int. 2015 [38]	100	2015	RCT	10	I	Türkiye
82	Coetzee JC, Giza E, Schon LC, Berlet GC, Neufeld S, Stone RM, Wilson EL. Treatment of osteochondral lesions of the talus with particulated juvenile cartilage. Foot Ankle Int. 2013 [15]	97	2013	Case series	8	IV	United States
83	Hannon CP, Ross KA, Murawski CD, Deyer TW, Smyth NA, Hogan MV, Do HT, O'Malley MJ, Kennedy JG. Arthroscopic Bone Marrow Stimulation and Concentrated Bone Marrow Aspirate for Osteochondral Lesions of the Talus: A Case‐Control Study of Functional and Magnetic Resonance Observation of Cartilage Repair Tissue Outcomes. Arthroscopy. 2016 [47]	97	2016	Case–control	11	III	United States
84	Nam EK, Ferkel RD, Applegate GR. Autologous chondrocyte implantation of the ankle: a 2‐ to 5‐year follow‐up. Am J Sports Med. 2009 [73]	96	2009	Case series	6	IV	United States
85	Hangody L. The mosaicplasty technique for osteochondral lesions of the talus. Foot Ankle Clin. 2003 [44]	95	2003	Review article	4	V	Hungary
86	van Bergen CJ, de Leeuw PA, van Dijk CN. Treatment of osteochondral defects of the talus. Rev Chir Orthop Reparatrice Appar Mot. 2008 [107]	95	2008	Review article	6	V	Netherlands
87	Thompson JP, Loomer RL. Osteochondral lesions of the talus in a sports medicine clinic. A new radiographic technique and surgical approach. Am J Sports Med. 1984 [103]	94	1984	Case series	2	IV	Canada
88	Hunt SA, Sherman O. Arthroscopic treatment of osteochondral lesions of the talus with correlation of outcome scoring systems. Arthroscopy. 2003 [52]	94	2003	Case series	4	IV	United States
89	Meehan R, McFarlin S, Bugbee W, Brage M. Fresh ankle osteochondral allograft transplantation for tibiotalar joint arthritis. Foot Ankle Int. 2005 [67]	94	2005	Case series	5	IV	United States
90	Choi WJ, Kim BS, Lee JW. Osteochondral lesion of the talus: could age be an indication for arthroscopic treatment? Am J Sports Med. 2012 [11]	94	2012	Cohort study	7	III	South Korea
91	Giannini S, Buda R, Grigolo B, Vannini F, De Franceschi L, Facchini A. The detached osteochondral fragment as a source of cells for autologous chondrocyte implantation (ACI) in the ankle joint. Osteoarthritis Cartilage. 2005 [32]	93	2005	Case control	5	III	Italy
92	Cuttica DJ, Smith WB, Hyer CF, Philbin TM, Berlet GC. Osteochondral lesions of the talus: predictors of clinical outcome. Foot Ankle Int. 2011 [16]	93	2011	Case series	7	IV	United States
93	Shea MP, Manoli A 2nd. Osteochondral lesions of the talar dome. Foot Ankle. 1993 [95]	92	1993	Review article	3	V	United States
94	Shearer C, Loomer R, Clement D. Nonoperatively managed stage 5 osteochondral talar lesions. Foot Ankle Int. 2002 [96]	91	2002	Case series	4	IV	Canada
95	Hahn DB, Aanstoos ME, Wilkins RM. Osteochondral lesions of the talus treated with fresh talar allografts. Foot Ankle Int. 2010 [42]	91	2010	Case series	6	IV	United States
96	Kim YS, Park EH, Kim YC, Koh YG. Clinical outcomes of mesenchymal stem cell injection with arthroscopic treatment in older patients with osteochondral lesions of the talus. Am J Sports Med. 2013 [55]	91	2013	Cohort study	8	III	South Korea
97	Elias I, Jung JW, Raikin SM, Schweitzer MW, Carrino JA, Morrison WB. Osteochondral lesions of the talus: change in MRI findings over time in talar lesions without operative intervention and implications for staging systems. Foot Ankle Int. 2006 [24]	89	2006	Cohort study	5	III	United States
98	Millington SA, Grabner M, Wozelka R, Anderson DD, Hurwitz SR, Crandall JR. Quantification of ankle articular cartilage topography and thickness using a high resolution stereophotography system. Osteoarthritis Cartilage. 2007 [69]	89	2007	Laboratory study	5	IV	United States
99	Takao M, Uchio Y, Kakimaru H, Kumahashi N, Ochi M. Arthroscopic drilling with debridement of remaining cartilage for osteochondral lesions of the talar dome in unstable ankles. Am J Sports Med. 2004 [100]	88	2004	Cohort study	4	II	Japan
100	Han SH, Lee JW, Lee DY, Kang ES. Radiographic changes and clinical results of osteochondral defects of the talus with and without subchondral cysts. Foot Ankle Int. 2006 [43]	88	2006	Case series	5	IV	South Korea

Abbreviation: RCT, randomized controlled trial.

**Figure 2:**
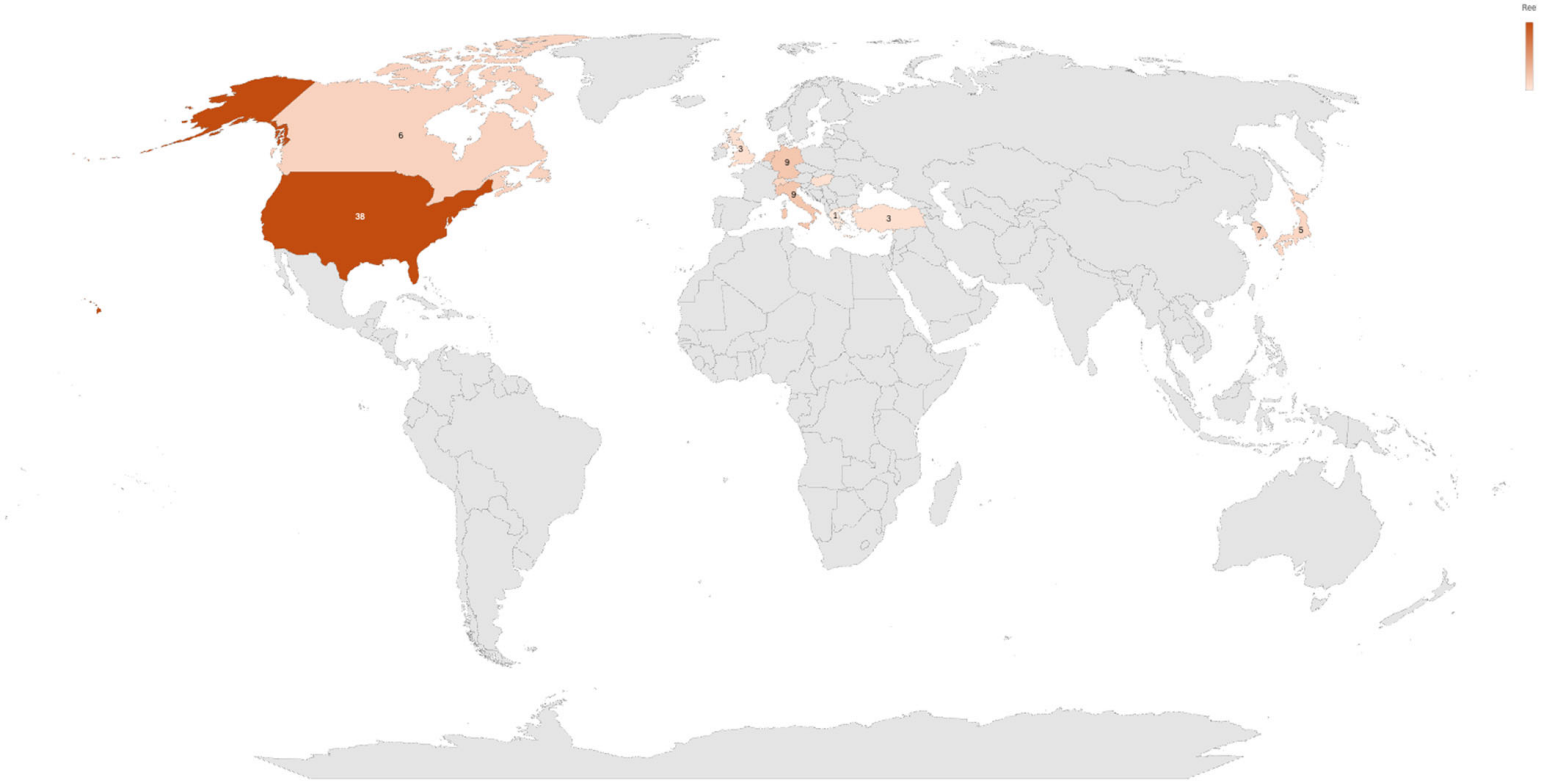
The percentage of the top 100 cartilage lesions of the ankle publications originating from each country. The included articles originated from 13 countries, led by the United States (*n* = 38), followed by Germany, Italy and the Netherlands (*n* = 9 each), South Korea (*n* = 7), Canada (*n* = 6), Japan and Switzerland (*n* = 5 each), Hungary, Turkey and the United Kingdom (*n* = 3 each), Israel (*n* = 2) and Greece (*n* = 1).

Articles were analyzed for the journal of publication and their country of origin. Overall, 19 different journals were represented. The journal with the most studies from the top 100 articles was the *American Journal of Sports Medicine*, with 25 citations. Furthermore, 81 of the 100 articles were published in five journals (Table 2). In total, these articles represented 13 different countries of origin. The United States represented 38 of the 100 articles, followed by the Netherlands, Italy and Germany with 9 articles each. South Korea followed with seven articles (Figure 2). When screening the top 100 citations by institution, the Department of Orthopedic Surgery and Sports Medicine from the University of Amsterdam scored the highest in terms of representation.

**Table 2:** The top five journals with the highest number of citations.

Rank	Journal	Number of articles
1	*American Journal of Sports Medicine*	25
2	*Foot and Ankle International*	23
3	*Journal of Bone and Joint Surgery – American Volume*	11
4	*Arthroscopy*	8
5 (shared) 5 (shared)	*Knee Surgery, Sports Traumatology, Arthroscopy* *The Bone and Joint Journal*	7 7
Total	81	

**Figure 3:**
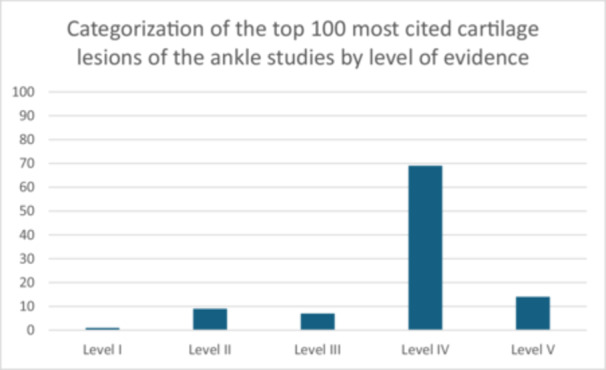
Categorization of the top 100 most‐cited articles on cartilage lesions of the ankle by level of evidence.

**Figure 4:**
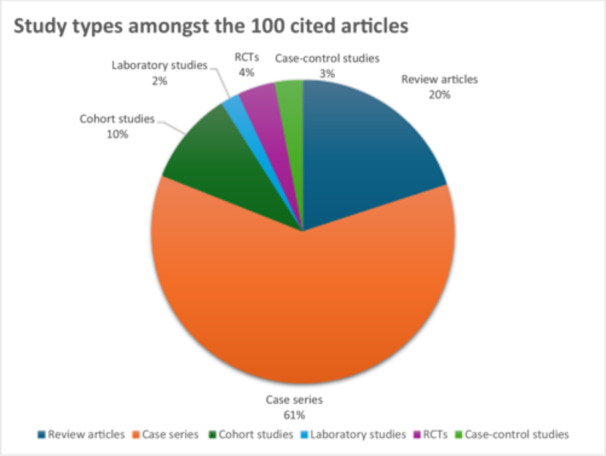
Percentage wise‐distribution of different study types among the top 100 most‐cited articles.

The levels of evidence included a single Level I study, 9 Level II studies, 7 Level III studies, 69 Level IV studies and 5 Level V studies (Figure 3). There were six different study types represented in the 100 articles (Figure 4).

Regarding research themes, the following outcomes were found: Of the 100 studies, 68 focused on treatment outcomes, 17 were reviews or overview articles, 0 addressed paediatric or juvenile OLTs, 11 primarily focused on radiology/imaging, and 4 investigated aetiology or pathophysiology. Citation density is described in Table 3.

**Table 3:** Top 10 articles with the highest citation density.

Rank	Article	Rank top 100 most cited	Citation density
1	Zengerink M, Struijs PA, Tol JL, van Dijk CN. Treatment of osteochondral lesions of the talus: a systematic review. Knee Surg Sports Traumatol Arthrosc. 2010 [116]	#1	33
2	Ramponi L, Yasui Y, Murawski CD, Ferkel RD, DiGiovanni CW, Kerkhoffs GMMJ, Calder JDF, Takao M, Vannini F, Choi WJ, Lee JW, Stone J, Kennedy JG. Lesion Size Is a Predictor of Clinical Outcomes After Bone Marrow Stimulation for Osteochondral Lesions of the Talus: A Systematic Review. Am J Sports Med. 2017 [83]	#26	27
3	Chuckpaiwong B, Berkson EM, Theodore GH. Microfracture for osteochondral lesions of the ankle: outcome analysis and outcome predictors of 105 cases. Arthroscopy. 2008 [14]	#3	23
3	Choi WJ, Park KK, Kim BS, Lee JW. Osteochondral lesion of the talus: is there a critical defect size for poor outcome? Am J Sports Med. 2009 [12]	#6	23
5	Hangody L, Vásárhelyi G, Hangody LR, Sükösd Z, Tibay G, Bartha L, Bodó G. Autologous osteochondral grafting‐‐technique and long‐term results. Injury. 2008 [46]	#4	22
**6**	Ferkel RD, Zanotti RM, Komenda GA, Sgaglione NA, Cheng MS, Applegate GR, Dopirak RM. Arthroscopic treatment of chronic osteochondral lesions of the talus: long‐term results. Am J Sports Med. 2008 [26]	#7	21
**6**	Raikin SM, Elias I, Zoga AC, Morrison WB, Besser MP, Schweitzer ME. Osteochondral lesions of the talus: localization and morphologic data from 424 patients using a novel anatomical grid scheme. Foot Ankle Int. 2007 [25]	#5	21
**8**	Dahmen J, Lambers KTA, Reilingh ML, van Bergen CJA, Stufkens SAS, Kerkhoffs GMMJ. No superior treatment for primary osteochondral defects of the talus. Knee Surg Sports Traumatol Arthrosc. 2018 [19]	#52	20
**9**	van Dijk CN, Reilingh ML, Zengerink M, van Bergen CJ. Osteochondral defects in the ankle: why painful? Knee Surg Sports Traumatol Arthrosc. 2010 [112]	#12	19
**10**	Murawski CD, Kennedy JG. Operative treatment of osteochondral lesions of the talus. J Bone Joint Surg Am. 2013 [72]	#23	18
**10**	O'Loughlin PF, Heyworth BE, Kennedy JG. Current concepts in the diagnosis and treatment of osteochondral lesions of the ankle. Am J Sports Med. 2010 [76]	#14	18
**10**	Mei‐Dan O, Carmont MR, Laver L, Mann G, Maffulli N, Nyska M. Platelet‐rich plasma or hyaluronate in the management of osteochondral lesions of the talus. Am J Sports Med. 2012 [68]	#22	18

## DISCUSSION

This bibliometric analysis of the 100 most‐cited articles on ankle cartilage lesions offers a comprehensive overview of the literature that influences this evolving field. The publications span multiple decades and reflect the progressive development of diagnostic methods and treatment strategies for OCLs of the ankle. It was hypothesized that the majority of the studies would have been published in the last 20 years; however, analyzing the data, the publication peak falls between 2008 and 2010, thereby rejecting the hypothesis. A significant proportion of these studies originate from the United States and predominantly consist of case series, representing a Level IV evidence base. This aligns with prior observations in orthopaedic literature; a similar finding was observed in the recent publication [77] on osteotomies of the knee as well as in a previous analysis on the level of evidence in ankle cartilage repair). Interestingly, citation frequency did not always correlate with publication age, suggesting that more recent innovations—such as advancements in biologics, arthroscopic techniques, and scaffold‐based treatment options technologies—may have generated rapid scholarly attention [87].

When analyzing the top 100 citations by research theme, it is apparent that more than two thirds of the publications in the 100 top‐cited articles consider treatment outcome publications, rather than overview or radiological studies. Additionally, when assessing the top 100 citations by department or hospital, the Department of Orthopedic Surgery and Sports Medicine at the University of Amsterdam emerged as the most represented. This trend is particularly notable when focusing on articles published after 2018, where manuscripts have had less time to accumulate citations. Notably, the most recent study among the top 100 also originated from this department [19], with several other recent high‐impact articles following closely, highlighting its continued influence in the field. Another important finding to be mentioned is that there were no paediatric studies included in the top 100 publications. The absence of paediatric studies among the most influential publications is therefore a noteworthy finding, and several factors may explain it. First, the overall volume of paediatric‐focused OLT research is considerably smaller than that available for the adult population, limiting the pool from which highly cited articles could emerge. Second, our search strategy and exclusion criteria may not have been sufficiently sensitive to capture this literature, particularly given that paediatric studies more often use alternative terminology such as ‘osteochondritis dissecans’ rather than ‘osteochondral lesion of the talus’. Third, dedicated paediatric OLT research remains a comparatively young field, with most large‐scale series and outcome studies published only within the past one to two decades, which may likely have too recent to have accrued citation counts comparable to older adult‐focused work.

The findings also underscore the importance of nuanced keyword selection in literature searches. Some influential articles did not explicitly include the term ‘cartilage lesion’ or ‘osteochondral defect’ in their titles, indicating that landmark research may be embedded within broader discussions or associated with combined procedures and combined injuries. These combined injuries may be instability of the ankle as well as malalignment of the ankle, and co‐existing lesions in patients with ankle fractures in which the main focus is on the ankle fracture rather than the chondral or osteochondral lesion. The finding that not all articles explicitly included the main search terms in their title highlights the necessity of a comprehensive search strategy when conducting systematic reviews or scoping literature reviews in this domain. It is noteworthy that the top 100 most‐cited studies are characterized by a paucity of high‐quality evidence, with a significant reliance on Level IV studies. As a result, treatment algorithms are primarily based on low‐quality evidence. This highlights a substantial research gap, underscoring the need for more high‐quality studies, particularly RCTs, to strengthen the evidence base. Additionally, it is important to note that the majority of studies in this top 100 most‐cited list originate from Western countries. This may be attributed to the inclusion criteria, which limited articles to those published in English. Consequently, this top‐100 list is susceptible to bias, as studies published in other languages, such as Japanese or Chinese, are excluded. Given the increasing research activity in ankle cartilage in these countries, it is important to consider the potential impact of their work, which may be underrepresented in this list.

The present study also includes a number of limitations due to the inherent methodological shortcomings of this bibliometric analysis. While citation count remains a widely accepted metric of academic impact, it should be noted that it also has inherent limitations. Articles may accrue citations due to author self‐referencing, institutional bias, or trends within specific journals. Furthermore, bibliometric analyses are subject to time bias, where older articles have had more opportunity to accumulate citations, and obliteration by incorporation, where seminal studies may be cited less frequently once their findings become widely accepted as common knowledge. Citation density (Table 3) is similarly an imperfect metric when comparing articles across different eras: a seminal paper from 1980 is effectively competing for citations against a 2015 paper within a scientific landscape where the overall volume of published output was vastly lower, meaning equal citation density does not necessarily reflect equal influence. An additional limitation relates to the use of a single database; citation counts were derived exclusively from Web of Science, and as citation coverage can vary substantially between Web of Science, Scopus, Google Scholar and Dimensions, the most‐cited list generated here may not fully overlap with one derived from an alternative or multi‐database search. High citation counts, therefore, do not always equate to high clinical relevance or methodological rigour. On the other hand, one could note that this bibliometric analysis offers several methodological strengths that enhance its reliability and relevance. First, it provides an objective, quantitative overview of the most‐cited publications within the field, based on citation data from a well‐established and widely used database. Second, by analyzing citation density in addition to total citation count, this study accounts for differences in publication age, helping to highlight more recent high‐impact articles that might otherwise be underrepresented. This dual metric approach balances long‐term influence with current relevance. Third, the inclusion of multiple reviewers for article selection and data extraction reduced the risk of selection bias and ensured consistency and accuracy in identifying eligible studies. Discrepancies were resolved through consensus, enhancing the methodological rigour of the analysis.

Regarding the clinical relevance of the present study, the present bibliometric analysis provides valuable insight into the most‐cited research shaping current understanding and management of ankle cartilage injuries. By identifying key articles, authors and specific topics, clinicians involved in the field of cartilage lesions of the ankle are provided with a comprehensive foundation summarizing the key literature on cartilage lesions of the ankle. For practitioners, this collection of top‐cited works offers a focused resource for guiding evidence‐based practice and enhancing patient care in this context. This analysis offers valuable insight into the level of evidence and study designs that have historically shaped clinical decision‐making in this area, both enabling an understanding of the evidentiary foundation of current practices and identifying areas where higher‐quality or prospective research is needed.

## CONCLUSION

The 100 most‐cited publications in cartilage lesions of the ankle were cited a total of 17,208 times. Case series and cohort studies were among the most frequently used study designs, with an overall low level of evidence, highlighting the need for higher‐quality evidence to strengthen the evidence base. Citation impact, however, does not necessarily equate to methodological quality, and this should be considered when interpreting citation frequency as a marker of clinical validity. The present work may function as a reference standard to help clinicians target future research directions.

## AUTHOR CONTRIBUTIONS

**Jari Dahmen**: Conceptualization; methodology; formal analysis; investigation; writing—original draft preparation; writing—review and editing. **Juliëtte H. M. Pijnacker**: Methodology; formal analysis; investigation; writing—original draft preparation; writing—review and editing. **Youichi Yasui**: Methodology; formal analysis; investigation; writing—original draft preparation; writing—review and editing. **Faridi S. Jamaludin**: Methodology; formal analysis; investigation; writing—original draft preparation; writing—review and editing. **Masato Takao**: Methodology; formal analysis; investigation; writing—original draft preparation; writing—review and editing. **John G. Kennedy**: Methodology; formal analysis; investigation; writing—original draft preparation; writing—review and editing. **Gino M. M. J. Kerkhoffs**: Conceptualization; methodology; formal analysis; investigation; writing—original draft preparation; writing—review and editing; resources; supervision. **International Society for Cartilage Repair of the Ankle**: Methodology; writing—original draft preparation; writing—review and editing.

## FUNDING INFORMATION

The authors have no funding to report.

## CONFLICT OF INTEREST STATEMENT

The authors declare no conflicts of interest.

## ETHICS STATEMENT

Due to the public nature of the exploited data, an institutional review board was not necessary for conduction of this study.

## Data Availability

The authors have nothing to report.
